# Prediction of Potential Habitat Distributions and Climate Change Impacts on the Rare Species *Woonyoungia septentrionalis* (Magnoliaceae) in China Based on MaxEnt

**DOI:** 10.3390/plants14010086

**Published:** 2024-12-30

**Authors:** Weihao Yao, Zenghui Wang, Yu Fan, Danyang Liu, Zeyang Ding, Yumei Zhou, Shuyue Hu, Wei Zhang, Jing Ou

**Affiliations:** 1 College of Forestry, Guizhou University, Guiyang 550025, China; ywh19831027397@163.com (W.Y.); 17385653247@163.com (Z.W.); gzufanyu@163.com (Y.F.); 13368628801@163.com (D.L.); zym21198508@163.com (Y.Z.); 15180870742@163.com (S.H.); zhangwei20201108@163.com (W.Z.); 2 College of Agricultural, Guizhou University, Guiyang 550025, China; dzy991217@sina.com

**Keywords:** *Woonyoungia septentrionalis*, MaxEnt, suitable habitat, influencing factors, migration pathways

## Abstract

Changes in species’ habitats provide important insights into the effects of climate change. *Woonyoungia septentrionalis*, a critically endangered species endemic to karst ecosystems, has a highly restricted distribution and is a key biological resource. Despite its ecological importance, the factors influencing its habitat suitability and distribution remain poorly understood. This study employed ecological niche modeling to predict the potential distribution of *Woonyoungia septentrionalis* across China and analyzed shifts in centroid location to explore migration pathways under current and future climate scenarios. The model exhibited high predictive accuracy (AUC = 0.988), indicating its robustness in assessing habitat suitability. Under current climatic conditions, *Woonyoungia septentrionalis* is predominantly found in the Guizhou–Guangxi border region, southeastern Yunnan, eastern Sichuan, southeastern Tibet, and parts of Chongqing, Hunan, and Hubei. Among these, the Guizhou-Guangxi border represents the primary suitable habitat. Temperature factors, particularly bio6 (minimum temperature of the coldest month) and bio7 (annual temperature range), were the most significant determinants of habitat suitability, contributing 43.29% and 12.65%, respectively. Soil cation exchange capacity (CEC) accounted for 15.82%, while precipitation had a relatively minor impact. Under future climate scenarios, suitable habitats for *Woonyoungia septentrionalis* are projected to shrink and shift toward higher altitudes and latitudes, increasing the risk of extinction due to the “mountain trap” effect, where migration is constrained by limited habitat at higher elevations. Stable habitats, particularly in Libo (Guizhou) and Huanjiang (Guangxi), are identified as critical refugia. We recommend prioritizing shrinking and stable habitats in Guizhou, Guangxi, and Yunnan for in situ conservation. Ex situ conservation efforts should focus on areas identified based on key environmental factors and predicted migration pathways to ensure the species’ long-term survival. This study provides both theoretical and practical guidance for the conservation of this species and its vulnerable habitat.

## 1. Introduction

Climate change profoundly influences species distribution and remains a cornerstone of ecological research [1]. In biogeography, understanding the complex interplay between plants and climate has become a critical area of study [2]. Evidence shows that climate change destabilizes species habitats [3], triggering cascading effects on ecosystem function, community composition, and the growth and spatial distribution of plant species [4]. Endangered and narrowly distributed species are particularly vulnerable, as habitat shifts may alter their ecological traits [5], compel range migrations to climatically suitable areas [6], or lead to the extinction of species unable to adapt to rapidly changing conditions [7]. Addressing these challenges requires predictive studies on habitat distribution under diverse climate scenarios, identifying the key environmental drivers shaping species distribution and forecasting shifts in habitat suitability. Such efforts are essential for developing conservation strategies tailored to rare and endangered species. By integrating predictive ecological models with targeted conservation planning, this research provides a robust scientific foundation for biodiversity conservation, species reintroduction, and domestication efforts, ensuring the persistence of vulnerable species in an era of rapid environmental change.

Species Distribution Models (SDMs), also known as habitat suitability or ecological niche models, are essential tools for assessing species’ ecological requirements and predicting suitable habitats by linking occurrence data with environmental variables [8,9]. Widely applied in plant conservation, habitat predictions, and managing biological invasions, SDMs are pivotal in protecting rare and endangered species [10]. Among commonly used models, namely ENFA, MaxEnt, GLM, GAM, and BIOCLIM [11,12], MaxEnt is particularly noted for its robustness and accuracy, especially under limited data conditions [13]. For example, MaxEnt has effectively forecasted the distribution and habitat shifts in rare species such as *Tetracentron sinense* [14], *Cyclobalanopsis gilva* [15], and *Ormosia microphylla* [16] under future climate scenarios. These findings provide valuable insights into species’ ecological requirements and distribution patterns, offering a scientific foundation for biodiversity conservation, habitat restoration, and adaptive management strategies in response to climate change.

*Woonyoungia septentrionalis* (Dandy) Y. W. Law (syn. *Kmeria septentrionalis*), an endemic species of the family Magnoliaceae, is native to China. This dioecious evergreen tree has been classified as a National Class I Protected Plant since 1999 and was assessed as Vulnerable (VU) on the IUCN Red List in 2017 [17]. In 2012, it was prioritized in the national conservation plan addressing 120 extremely small populations. The species primarily inhabits karst ecosystems, with significant populations concentrated in the Maolan Nature Reserve (Guizhou) and the Mulun Nature Reserve (Guangxi), and smaller, fragmented populations were reported in Maguan and Hekou (Yunnan Province). The species’ straight, tall trunk rendered it a highly sought-after timber resource, resulting in extensive logging that severely degraded its natural habitats. These anthropogenic pressures, compounded by its limited natural regeneration capacity, have placed *W. septentrionalis* under severe threat in the wild. The species was first described as *Kmeria septentrionalis* by British botanist J.E. Dandy in 1931, representing its formal introduction to the scientific community. However, habitat destruction at its type locality led to its presumed extinction, until its rediscovery in 1987 by Liu Yuhu and colleagues in Huanjiang (Guangxi) and Libo (Guizhou). In 1997, it was reclassified as *W. septentrionalis*, becoming the type species of a newly established genus [18]. Subsequent taxonomic revisions in 2005 by Lin Qi and colleagues synonymized *W. septentrionalis* and *Magnolia kwangsiensis* under *Kmeria septentrionalis*, effectively rendering *Woonyoungia* a synonym. Further research in 2008 investigated its floral morphology, revealing that its pistillate flowers likely evolved from reduced bisexual flowers [19], thus providing valuable insights into its evolutionary history and reproductive adaptations.

Studies by Xu Fengxia [20], Xi Yizhen [21], and their colleagues on the pollen and exine structure have provided critical insights into the morphological and evolutionary significance of *W. septentrionalis*. Zeng Qingwen [22] examined flower number and positioning, offering essential data that supported the establishment of the *Woonyoungia*. Lai Jiaye [23,24] and colleagues analyzed floral traits and pollen dispersal, uncovering that floral specialization is an adaptation to extended rainfall during the flowering period. The lack of effective pollinators and competition from co-flowering species are key factors contributing to its endangered status. Qin Wengeng [25] explored the relationship between flowering phenology and meteorological factors, concluding that rainfall and relative humidity delay flowering onset. Tan Jingui [26] investigated reproductive biology, finding that male gametophyte degeneration is not a primary threat to its survival. Jin Junyan [27,28] and Wang Guohai [29] examined population ecology, identifying rock exposure, canopy cover, and soil moisture as primary factors influencing its distribution. The species’ clustered distribution is shaped by both seed biology and karst habitat characteristics. In the Mulun Nature Reserve, *W. septentrionalis* plays a dominant ecological role, with community succession supporting its growth. Peng Yuhua [30,31] studied soil characteristics and spatial structure, identifying the optimal nutrient ranges for growth. Despite these contributions, the species’ weak natural regeneration remains a significant threat to its long-term viability. However, research on its habitat suitability and the environmental drivers of its distribution is still limited. Therefore, further investigation into the impacts of climate change, habitat preferences, and population dynamics is crucial for developing effective conservation strategies.

This study investigates the potential suitable habitat of *W. septentrionalis* in China by integrating geographical distribution data with tools such as MaxEnt, ArcGIS10.2, and R4.3.2. The objectives are to model the species’ current habitat distribution, identify key environmental factors using the jackknife method, and evaluate habitat dynamics under future climate change scenarios. By analyzing distribution patterns and environmental drivers, the study aims to clarify the relationship between species distribution and environmental variables. These findings will support the development of targeted conservation strategies and provide a scientific basis for the species’ introduction, sustainable utilization, and management.

## 2. Materials and Methods

### 2.1. Occurrence Data Collection

**Figure 1:**
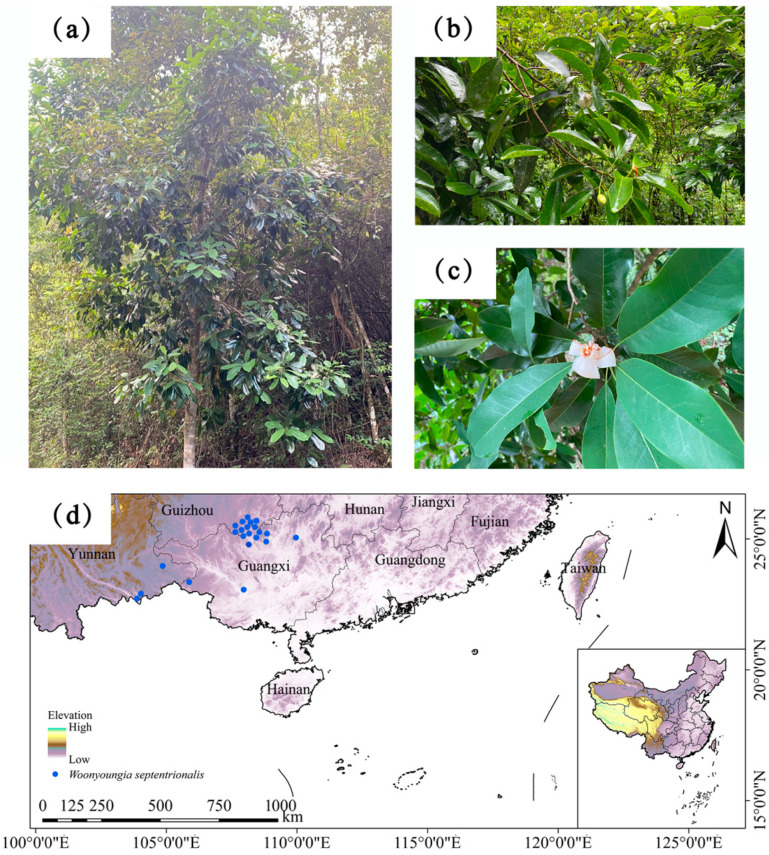
Photographs (**a**–**c**) and occurrence records (**d**) of *W. septentrionalis* used in the MaxEnt model. All photographs were taken by the author.

Species distribution data for *W. septentrionalis* were obtained from two primary sources: (1) field surveys, which recorded 43 distribution points, and (2) herbarium specimens, online databases, and the literature, including the Chinese Virtual Herbarium (CVH, https://www.cvh.ac.cn/, accessed on 25 June 2024) and the Global Biodiversity Information Facility (GBIF, https://www.gbif.org/zh/, accessed on 25 June 2024), as well as journals and books, contributing an additional 89 records. To reduce overfitting risks, distribution data were filtered using ENM_Tools v1.3 [32]. The software automatically adjusts the grid size of environmental factors, removes redundant data within the same grid cell, and ensures that only one valid distribution point is retained per grid [33]. Within 30″ grid cells (approximately 1 km × 1 km), only one distribution point was retained. After processing, 48 samples distribution points were retained, stored in a CSV file for MaxEnt model construction, and are visualized in Figure 1.

### 2.2. Source and Selection of Environmental Data

This study incorporates environmental data, including climatic, topographic, and soil variables, totaling 32 factors. Specifically, 19 bioclimatic variables (bio1–bio19) and 3 topographic variables (elevation, slope, and aspect) were obtained from WorldClim (https://www.worldclim.org/, accessed on 25 June 2024), representing current conditions (1970–2000) and future scenarios for the 2050s (2041–2060) and 2070s (2061–2080). Future projections were based on the BCC-CSM2-MR climate model under three Shared Socioeconomic Pathways (SSP126, SSP245, and SSP585) at a spatial resolution of 30″ [34,35]. Nine soil variables were derived from the Harmonized World Soil Database (HWSD, https://www.fao.org/soils-portal/data-hub/en/, accessed on 25 June 2024) (Table 1). All variables were standardized using ArcGIS 10.2, reprojected to the WGS 1984 geographic coordinate system, and aligned with administrative boundaries to ensure compatibility and precision in spatial analyses. This preprocessing facilitated robust modeling and spatial interpretation of the environmental constraints affecting *W. septentrionalis* distribution.

To mitigate multicollinearity among climate variables and prevent model overfitting, a systematic approach was employed. Initially, a pre-run of the MaxEnt model was conducted to assess the importance and contribution of environmental variables using a Jackknife test [36]. Environmental data were then sampled using the extraction tool in ArcGIS, and Pearson correlation coefficients among the variables were calculated. Variables with Pearson’s correlation coefficient |r| > 0.85 were screened, and variables contributing zero to the predictive power of the model were excluded [37]. This process ensured that only the environmental variables most closely associated with species distribution were retained, optimizing the final selection of predictor variables [38,39]. Ultimately, from an initial set of 32 environmental factors, 9 climatic variables (bio6, bio7, bio8, bio9, bio14, bio15, bio16, bio18, and bio19), 2 soil variables (t_cec_soil, t_cec_clay), and 1 topographic variable (elevation) were selected for the final model [40] (Table 2).

### 2.3. Model Parameter Optimization

**Figure 2:**
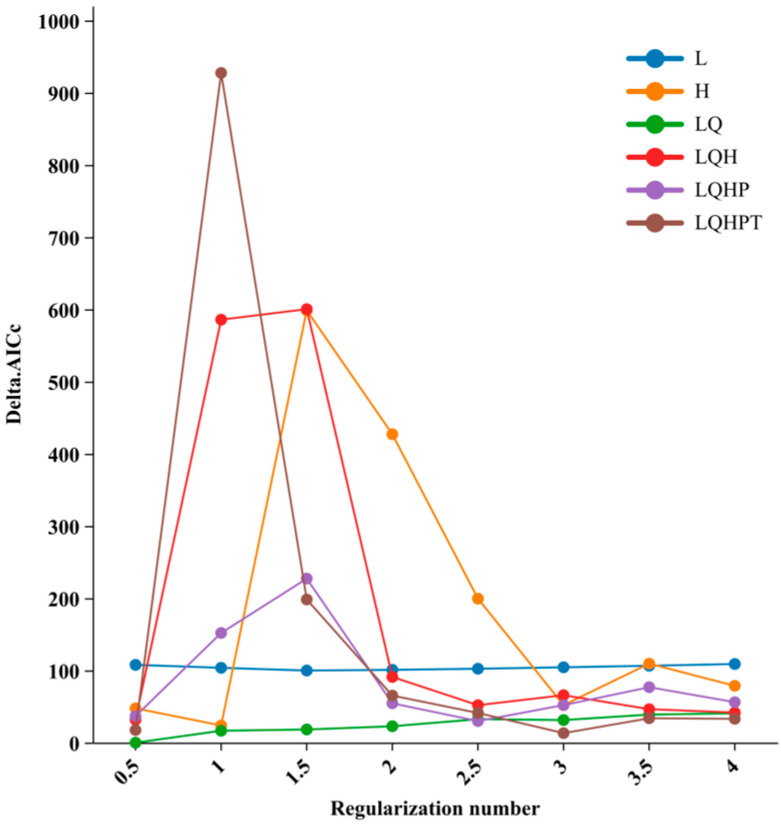
Performances of MaxEnt model in simulating and predicting the potential distribution of *W. septentrionalis* in different settings.

The parameter settings of the MaxEnt model significantly influence the precision of environmental sampling and the sensitivity of predictions, which in turn affect model transferability and accuracy. In this study, we optimized the regularization multiplier (RM) and feature combination (FC) parameters using five default feature types: linear (L), quadratic (Q), hinge (H), product (P), and threshold (T). The ENMeval package in R version 4.3.2 was utilized to optimize and fine-tune the MaxEnt model [41]. RM parameters were tested at intervals of 0.5 across a range from 0 to 4 and cross-validated with different FC combinations. Model parameters were selected to balance low complexity and high transferability, following established criteria [38]. Model selection was guided by the Akaike Information Criterion corrected for small sample sizes (AICc), where lower delta AICc values indicated better predictive performance [42]. Figure 2 illustrates the model selection process and results.

### 2.4. Model Establishment and Accuracy Assessment

**Figure 3:**
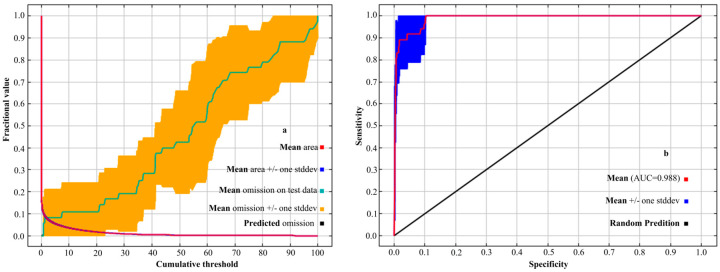
The validation of the MaxEnt model predicting *W. septentrionalis* distribution: (**a**) omission rate and (**b**) ROC curve.

Distribution data for 48 samples and 12 environmental variables were incorporated into the MaxEnt model, with 75% of the data used for model training and 25% for testing. Single-factor response curves were generated to assess species’ responses to environmental factors. A probability threshold of *p* ≤ 0.5 indicated limited ecological suitability, whereas *p* > 0.5 indicated favorable suitability [43]. Species distribution maps were generated, and the significance and relative contributions of each environmental variable were assessed using Jackknife resampling. The regularization multiplier (RM) and feature combination (FC) parameters were optimized according to model performance criteria, while other parameters were maintained at their default values. This process was repeated ten times to ensure consistency and robustness in the model results. Model accuracy was assessed using Receiver Operating Characteristic (ROC) curves, which are robust to variations in sample size and threshold settings, making them particularly suitable for evaluating MaxEnt model performance. Figure 3 displays the AUC (Area Under Curve) values for the training set, ranging from 0.5 to 1.0, with higher values indicating greater model accuracy [13,44]. According to AUC thresholds, values between 0.5 and 0.6 represent poor model performance, from 0.6 to 0.7 to be fair, from 0.7 to 0.8 to be good, from 0.8 to 0.9 to be very good, and from 0.9 to 1.0 to be excellent [45].

### 2.5. Classification and Dynamic Changes in Habitat Suitability Zones

The output results of the MaxEnt model were visualized using the reclassification tool in ArcGIS. Suitability levels were classified based on methods used for Magnoliaceae species, karst-endemic plants, and rare plants. Habitat suitability was divided into four levels using a manual classification approach: unsuitable (0–0.1), low (0.1–0.3), moderate (0.3–0.5), and high suitability (0.5–1.0) [32]. Suitable habitat areas for *Woonyoungia septentrionalis* were calculated across different time periods, and changes in distribution were analyzed under future climate scenarios. Areas with a species suitability index (SSI) ≥ 0.1 were designated as suitable habitats, whereas those with SSI < 0.1 were classified as unsuitable. A binary presence–absence matrix (0, 1) was generated to denote the presence (1) or absence (0) of suitable habitats for *Woonyoungia septentrionalis* under future climate scenarios. Using this matrix, changes in suitable habitat distribution under different scenarios were categorized into four types: unsuitable, degraded, persistent (original suitable), and newly suitable areas [46].

### 2.6. Species Distribution Centroid Migration Routes

The direction and distance of habitat shifts for *W. septentrionalis* were determined by analyzing changes in the positions of geometric centroids, a method commonly used to quantify spatial distribution dynamics [47]. The SDM_Toolbox in ArcGIS 10.2 was used to extract geometric centroids from the binary outputs of current and projected suitable habitats [32]. These tools facilitated habitat classification and centroid determination. Coordinates of the centroids were then used to calculate positional shifts and migration distances of *W. septentrionalis* under different climate change scenarios [48].

## 3. Results and Analysis

### 3.1. Optimal Model and MaxEnt Model Evaluation

As shown in Figure 2, the initial MaxEnt model with default parameters (RM = 1, FC = LQHPT) yielded a delta AICc value of 927.48, indicating suboptimal performance. To improve model performance and minimize the delta AICc value, the regularization multiplier (RM) was adjusted to 0.5 and the feature combination (FC) was simplified to LQ, which reduced model complexity while maintaining predictive capability. Figure 3 presents the Receiver Operating Characteristic (ROC) curve, where the Area Under Curve (AUC) achieved a value of 0.988. This high AUC value demonstrates the model’s excellent predictive performance, reflecting both accuracy and reliability. The optimized model successfully met the study’s precision criteria, providing robust predictions of suitable habitat distribution.

### 3.2. Contribution Assessment of Environmental Variables

**Figure 4:**
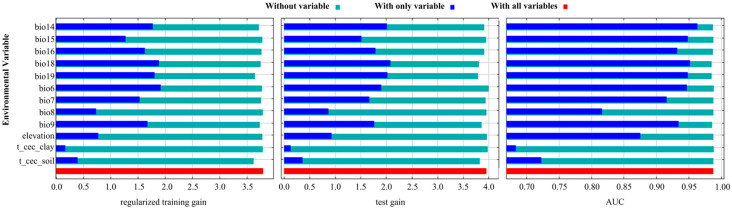
Jackknife test for environmental variables.

Table 2 presents the variables contributing more than 10% to the analysis, namely bio6, t_cec_soil, and bio7, which account for 43.29%, 15.82%, and 12.65%, respectively. In the permutation importance analysis, bio6 and bio7 demonstrated greater significance, with contributions of 26.77% and 23.28%, respectively. Figure 4 further illustrates the relative importance of bio18, bio6, bio19, and bio14. Following screening, six dominant factors were identified, comprising two temperature variables, three precipitation variables, and one soil variable, collectively contributing 84.03%. Notably, bio6 had the largest influence (43.29%), followed by t_cec_soil (15.82%) and bio19 (6.92%). The temperature variables (bio6 and bio7) had a greater contribution than the soil (t_cec_soil) and precipitation variables (bio14, bio18, and bio19). In conclusion, the key factors influencing the distribution of *W. septentrionalis* are bio6, t_cec_soil, bio7, bio19, bio14, and bio18, with temperature variables—particularly bio6—playing the most significant role.

### 3.3. Dominant Environmental Variables Response Range

**Figure 5:**
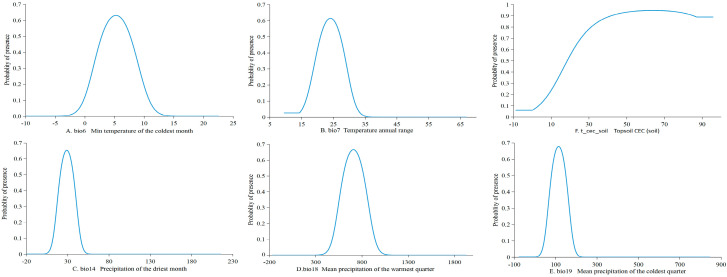
Response curves of *W. septentrionalis* to important climatic and edaphic factors.

Figure 5 illustrates the optimal survival thresholds for *W. septentrionalis* across various environmental factors. For the minimum temperature of the coldest month (bio6), the probability of presence is greater than 0.5 when temperatures range from 2.8 to 7.6 °C, peaking at 0.63 at 5.3 °C. The probability of presence remains greater than 0.5 for the annual temperature range (bio7) between 21 °C and 27 °C, with a peak of 0.61 at 24 °C. For topsoil CEC (t_cec_soil), the probability of presence is greater than 0.5 when values exceed 18.5 cmol/kg, peaking at 0.95 at 57 cmol/kg, after which it stabilizes. For precipitation in the driest month (bio14), the probability of presence is greater than 0.5 when precipitation ranges from 21 to 36.7 mm, with a peak suitability probability of 0.65 at 28.8 mm. For mean precipitation in the warmest quarter (bio18), the probability of presence exceeds 0.5 when precipitation is between 595 mm and 820 mm, with a peak suitability probability of 0.67 at 707 mm. For the coldest quarter (bio19), the probability of presence exceeds 0.5 when precipitation ranges from 79.5 mm to 150 mm, with a peak suitability probability of 0.68 at 110 mm. In summary, the optimal habitat for *W. septentrionalis* is formed when the minimum temperature of the coldest month (bio6) is 5.3 °C, the annual temperature range (bio7) is 24 °C, topsoil CEC (t_cec_soil) is 57 cmol/kg, precipitation in the driest month (bio14) is 28.8 mm, mean precipitation in the warmest quarter (bio18) is 707 mm, and mean precipitation in the coldest quarter (bio19) is 110 mm.

### 3.4. Current and Future Potential Distribution of W. septentrionalis in China

**Figure 6:**
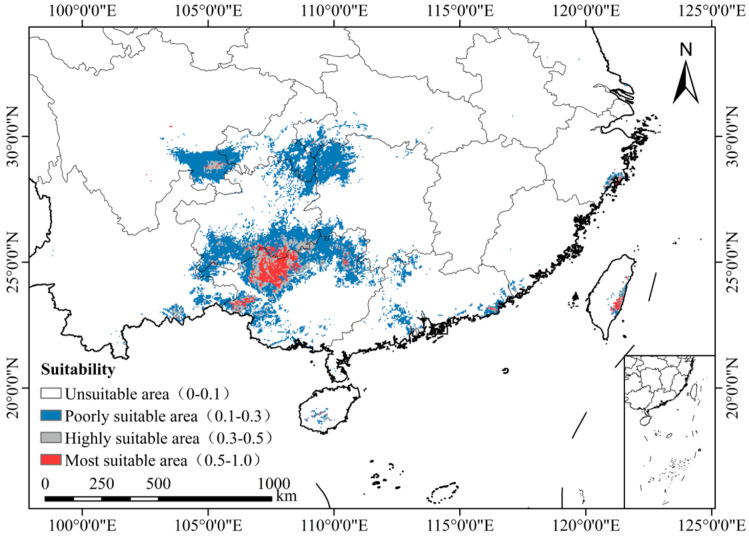
Distribution of suitable areas of *W. septentrionalis* under current climatic conditions.

**Figure 7:**
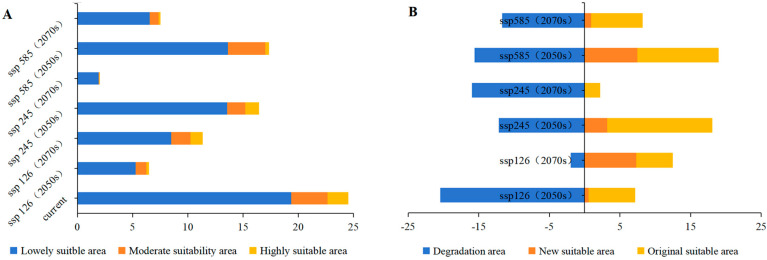
Areas (**A**) and area changes (**B**) of predicted suitable habitat in different climatic periods based on MaxEnt model.

Under current conditions, the total suitable habitat area of *W. septentrionalis* is 24.526 × 10^4^ km^2^, with 1.883 × 10^4^ km^2^ classified as highly suitable, 3.265 × 10^4^ km^2^ as moderately suitable, and 19.378 × 10^4^ km^2^ as insignificantly suitable (Figure 6 and Figure 7A).

**Figure 8:**
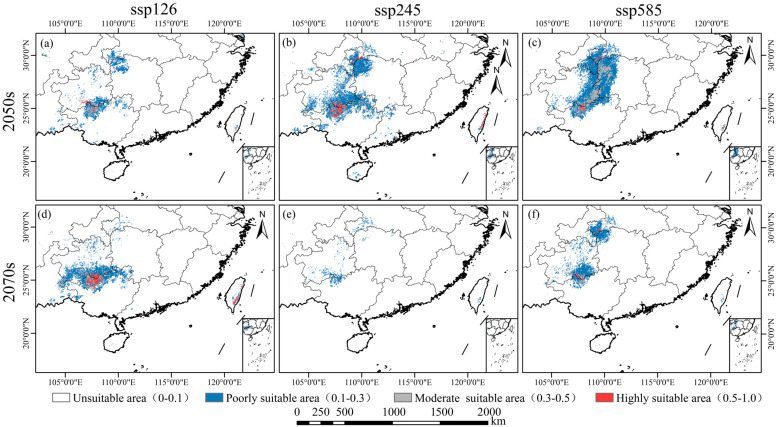
Map of predicted distribution of suitable areas. Predicted distribution under SSP126 scenario in the 2050s (**a**), 2070s (**d**), under the SSP245 scenario in the 2050s (**b**), 2070s (**e**), under the SSP585 scenario in the 2050s (**c**), and 2070s (**f**).

Figure 7A and Figure 8 illustrate future scenarios, revealing significant changes in the distribution of suitable habitats. All future scenarios predict a decline in suitable habitats compared to current conditions. By 2050, reductions in habitat area differ across the scenarios. Under SSP126, the habitat area decreases most substantially, totaling 6.489 × 10^4^ km^2^, with only 0.241 × 10^4^ km^2^ classified as highly suitable. Under SSP245, the habitat area totals 16.455 × 10^4^ km^2^, with 1.235 × 10^4^ km² classified as highly suitable. Under SSP585, the habitat area totals 17.360 × 10^4^ km², with 0.349 × 10^4^ km² classified as highly suitable. By 2070, only SSP126 exhibits an expanding trend, with a total habitat area of 11.343 × 10^4^ km^2^, of which 1.103 × 10^4^ km^2^ is highly suitable. In contrast, SSP245 exhibits the largest reduction, with a total habitat area of 1.985 × 10^4^ km² and only 0.010 × 10^4^ km^2^ classified as highly suitable. SSP585 exhibits a smaller reduction, with a total habitat area of 7.538 × 10^4^ km², of which 0.177 × 10^4^ km² is highly suitable. *W. septentrionalis* is currently primarily distributed in Libo (Guizhou) and Huanjiang (Guangxi). Despite future habitat changes under different scenarios, the main suitable areas for *W. septentrionalis* are expected to remain relatively stable.

### 3.5. Future Ecological Dynamics of W. septentrionalis

**Figure 9:**
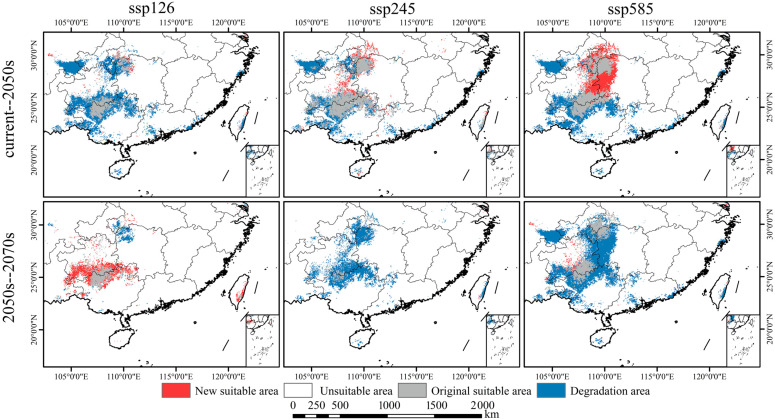
Spatial change patterns of suitable habitats of *W. septentrionalis* under different climate scenarios.

As illustrated in Figure 7B and Figure 9, projected future climate scenarios suggest significant shifts in the suitable habitat distribution for *W. septentrionalis*, with distinct patterns across different time points and scenarios. By 2050, all scenarios predict an overall reduction in suitable habitat relative to current conditions, although the extent of habitat loss and retention varies. Under the ssp126 scenario, the suitable habitat is projected to experience the greatest decline, with an 83.39% reduction, retaining only 26.97% of the current area. Conversely, the ssp245 scenario shows the least reduction, with a 49.56% loss and 60.80% of the current area remaining intact. The ssp585 scenario indicates a moderate reduction of 63.65%, with 46.71% of the habitat stable, and predicts the largest potential expansion of newly suitable areas. By 2070, only the SSP126 scenario shows an expansion trend in suitable habitats, while all other scenarios indicate contraction. Under SSP126, the area of newly suitable habitat is the largest, with minimal reduction, as contracted areas account for 30.41% of the 2050 suitable habitat and stable areas comprise 79.78%. In contrast, SSP245 displays the smallest increase in new suitable areas and the highest contraction, as contracted areas account for 96.99% of the 2050 suitable habitat and stable areas comprise 12.90%. The SSP585 scenario provides the highest stability, showing a moderate reduction trend, where contracted areas constitute 67.26% of the 2050 habitat and stable areas account for 41.88%. Under future climate scenarios, the suitable habitat of *W. septentrionalis* is expected to diminish in Yunnan, Sichuan, and Taiwan, while potential expansions are projected in Guizhou and Guangxi. Notably, stable habitat zones are likely to persist in Libo (Guizhou) and Huanjiang (Guangxi), albeit with potential slight shifts in suitability levels.

### 3.6. Centroid Migration Analysis

**Figure 10:**
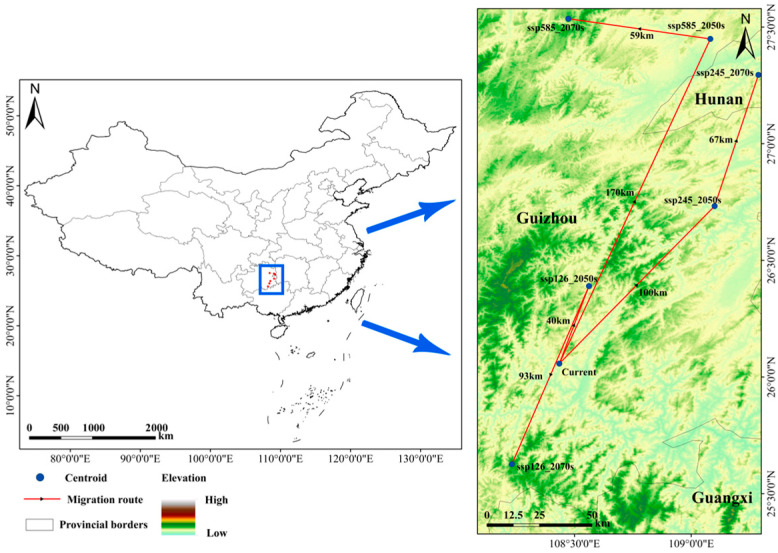
The centroid change in suitable habitats of *W. septentrionalis* under different climate scenarios.

Figure 10 illustrates the current centroid of *W. septentrionalis*’ suitable distribution, located in Rongjiang County, Guizhou Province. Under the SSP126 scenario, it is projected that, by 2050, the centroid will shift northward by 40 km, remaining within Rongjiang County. By 2070, the centroid is expected to move southward by 93 km to Libo County, Guizhou Province. Under the SSP245 scenario, by 2050, the centroid will migrate northward by 100 km to Tianzhu County, Guizhou Province, and by 2070, continue northward by 67 km to Xinhua County, Hunan Province. Under the SSP585 scenario, by 2050, the centroid will move northward by 170 km to Yuping County, Guizhou Province, and by 2070, shift westward by 59 km to Shiqian County, Guizhou Province. In 2050, slight variations in the centroid’s shift across different climate scenarios suggest a general northward trend. By 2070, however, significant differences emerge in migration patterns: a southward shift under SSP126, a continued northward shift under SSP245, and a northwestward shift under SSP585.

## 4. Discussion

### 4.1. Model Prediction Accuracy

The accuracy of the MaxEnt model depends on species distribution data and environmental variables, which are essential for estimating climatic and ecological requirements. The model’s predictions visualize species distribution patterns under varying temporal and climatic scenarios [36]. Previous studies show that model accuracy is highly sensitive to the selection of distribution data and environmental variables; excessive or inaccurate data can introduce errors [49]. The model performs optimally with more than five records and stabilizes with approximately 120 occurrences. The model’s default parameters, derived from simulations with 226 species across six regions [50], can be optimized for different species to improve accuracy [51]. To minimize sampling bias, buffer zones were applied to filter distribution data and reduce spatial overlap. To prevent overfitting from multicollinearity [36], correlation analysis, environmental variable contribution, and Jackknife resampling were used to select key variables. The ENMeval package optimized parameters, with RM = 0.5 and FC = LQ providing the best fit. Model accuracy was evaluated using the ROC curve, with the habitat suitability model for *W. septentrionalis* achieving an AUC of 0.988. This result is consistent with the predictive accuracies of other species, such as *Michelia martini* [52], *Parakmeria* [53], *Manglietia pachyphylla* [54], *Pinus* [55], and Cupressaceae [56], demonstrating strong performance in predicting suitable habitats with high accuracy and reliability [36].

### 4.2. Environmental Factors Influencing Habitat Distribution

The geographic distribution of plant species is fundamentally influenced by environmental factors, which mediate interactions between species and their habitats and ultimately determine spatial distribution patterns. Habitat suitability, quantified through ecological models, encapsulates these relationships by linking species occurrences with environmental conditions. In this study, six key factors influencing the habitat suitability of *W. septentrionalis* were identified using the Jackknife test, variable contribution analysis, and univariate response curves. Among these, the minimum temperature of the coldest month (bio6) emerged as the most critical factor, with temperature-related variables (bio6 and bio7) collectively contributing 55.94% to the model’s explanatory power. Soil properties, particularly the cation exchange capacity (t_cec_soil), accounted for 15.82%, while precipitation variables (bio14, bio18, and bio19) contributed 12.26%. These findings underscore the dominant role of temperature in shaping the distribution of *W. septentrionalis*.

Temperature and precipitation are widely recognized as critical determinants of the geographic distribution of Magnoliaceae species. Previous studies on rare species within this family, including *Parakmeria* [53], *Oyama sieboldii* [57], *Liriodendron chinense* [58], and *Manglietia insignis* [59], have consistently highlighted the influence of these climatic factors. The findings of this study align with these observations. *W. septentrionalis*, a dioecious plant species endemic to karst ecosystems, exhibits a narrow distribution range and pronounced sensitivity to environmental variables. Research on its biological traits and pollination ecology [23,24,25] reveals that temperature has a more pronounced impact on its survival and reproductive success compared to precipitation. This aligns with broader evidence that climatic variables, particularly temperature, critically influence seed germination, seedling establishment, and the distribution of rare and endangered species [60,61].

As a characteristic species of karst ecosystems, *W. septentrionalis* is primarily distributed in the karst regions of Yunnan, Guizhou, and Guangxi. The unique geological and climatic conditions of these areas heighten the species’ sensitivity to habitat changes. In karst habitats, extensive rock exposure and thin soil layers result in substantial soil temperature fluctuations which hinder the natural germination of *W. septentrionalis* seeds. However, temporary spring rainfall provides sufficient moisture for seed germination, highlighting the critical role of temperature in this process [62]. Soil factors, particularly t_cec_soil, also play a significant role in the species’ distribution by reflecting soil fertility. Analysis of soil characteristics within *W. septentrionalis* communities [63] has revealed elevated levels of organic matter, total nitrogen, available nitrogen, and effective zinc, all of which promote its growth. These findings validate the model’s accuracy and provide a robust scientific basis for the introduction, domestication, and ex situ conservation of *W. septentrionalis*.

### 4.3. Dynamic Changes in Habitat Suitability and Migration Routes of W. septentrionalis Distribution Centers

Using MaxEnt and GIS tools, this study analyzed species distribution and climatic data to predict the responses of *W. septentrionalis* under different temporal and climate change scenarios, highlighting its distinct ecological sensitivities. This study elucidated the spatial distribution patterns and projected the migration of potential distribution centers for *W. septentrionalis* under both current and future climate scenarios, offering valuable insights into conservation strategies and ecosystem management. The results indicate that the current geographic range of *W. septentrionalis* spans 105°51′–109°22′ E and 23°02′–26°08′ N, predominantly within southwestern China. This range includes southeastern Guizhou, northern and eastern Guangxi, southeastern Yunnan, and southeastern Sichuan, aligning closely with its observed distribution.

Future climate warming is anticipated to reshape species’ suitable habitats, intensifying habitat fragmentation and loss. This trend poses significant threats to habitat specialists, small populations, and species with limited natural regeneration capacity, consequently heightening extinction risks [30,64]. Species exhibit varied distribution patterns and evolutionary trends depending on their adaptive capacities to distinct environmental conditions [7]. Based on the investigation by Wang et al. into the role of bird foraging in the seed dispersal of *W. septentrionalis*, it was found that bird foraging enhances the probability of seed establishment in new habitats, promoting population regeneration. However, birds generally forage in secure environments, limiting their foraging range and, consequently, the distance over which seeds are dispersed [65]. Research on the role of rodents in seed handling revealed that most fallen seeds are consumed locally, reducing seed availability for regeneration rather than being relocated or stored. This local consumption thus limits both seed dispersal and germination of *W. septentrionalis* [66,67]. Studies on the effects of litter cover on seed germination show that while litter extends seed survival, it does not influence seed germination rates [68]. Further research by Wang on population regeneration in karst landscapes found that high rock exposure, nutrient-poor soils, and patchy soil distribution hinder population persistence. Additionally, steep slopes, combined with thick litter cover, restrict seed movement across the terrain, further limiting seed dispersal [69]. Pan et al.’s study on seed rain and natural regeneration revealed that prolonged seed dormancy exacerbates seed predation, significantly reducing seed availability and inhibiting seedling regeneration [70]. Moreover, the scarcity of middle-aged trees and the slow growth of seedlings contribute to poor population regeneration. These factors—limited seed dispersal, insufficient seedling recruitment, and a lack of middle-aged saplings—suggest potential habitat contraction for *W. septentrionalis* in the future, which aligns with the findings of this study.

The highly suitable habitats of *W. septentrionalis* in Guizhou and Guangxi are gradually diminishing and are currently concentrated in Libo and Huanjiang, particularly within Guizhou’s Maolan Nature Reserve and Guangxi’s Mulun Nature Reserve, which represent persistent suitable habitats under different periods and scenarios. These regions are hypothesized to serve as “local refugia” for *W. septentrionalis*, where the species maintains stable populations in karst habitats within and adjacent to protected areas. Such regions provide favorable microclimates and lower altitudes with adequate vertical migration space, supporting potential altitudinal shifts to higher elevations. Under future climate warming scenarios, the reduction in highly suitable habitats is expected to drive species toward upslope migration, resulting in decreased habitat availability and potentially creating an “elevation trap”. As warming continues, further upslope migration could constrain escape routes, leaving species unable to adapt to shrinking habitats and thereby increasing risks of local extinction [11,71].

By 2050, the suitable habitat centroids of *W. septentrionalis* are projected to shift northeastward under different climate scenarios. Migration distances are relatively shorter under ssp126 and ssp245 scenarios; however, under the ssp585 scenario, the centroid is expected to migrate up to 170 km northward, representing the most pronounced shift. This trend aligns with the broader migration patterns observed in temperate tree species, which tend to move toward higher latitudes and elevations under warming climates [72,73]. By 2070, these shifts become more pronounced, with centroids migrating southwestward to higher elevations under the ssp126 scenario, continuing northeastward under ssp245, and shifting northwestward to higher elevations under ssp585. These projections emphasize the critical role of temperature in shaping future distribution patterns, highlighting the necessity of prioritizing temperature factors alongside soil fertility and precipitation conditions in conservation strategies, species introduction, and cultivation planning.

Future climate warming is predicted to substantially impact the habitat suitability of *W. septentrionalis*, with low-latitude and low-elevation regions experiencing significant reductions in suitable areas, potentially leading to habitat loss. Southwest China, recognized as a biodiversity hotspot [74], origin center [75], distribution hub [76,77], and refuge for Magnoliaceae species [78], is identified as a critical diversification center [79]. Based on predictions of habitat suitability, migration routes, and current distribution, Libo and Huanjiang are hypothesized as key origin and distribution centers for *W. septentrionalis*. Validation of this hypothesis through molecular studies and fossil analysis is strongly recommended to provide further insights into the evolutionary history and biogeographic patterns of this species.

## 5. Conclusions

This study employed ecological niche modeling to evaluate the impact of climate warming on the geographic distribution of *W. septentrionalis*. The species is currently distributed primarily in the border regions of Guizhou, Guangxi, and southeastern Yunnan, with particularly favorable habitat conditions found in Libo (Guizhou) and Mulun (Guangxi). Projections under different future climate scenarios generally indicate habitat contraction; however, the SSP126 scenario predicts relatively favorable conditions, with an anticipated range expansion by 2070, suggesting its potential for long-term persistence. The centroid analysis further showed a gradual migration of *W. septentrionalis* toward higher latitudes and elevations due to climate warming. The environmental factor analysis identified temperature as the primary determinant of habitat suitability for the species. Further investigations of these environmental factors are essential to understanding the species’ adaptive strategies to climate warming, and will inform more effective management and conservation approaches. Future studies should integrate multiple modeling techniques, incorporate a broader range of environmental variables, and expand occurrence data to improve the accuracy of habitat suitability predictions.

## Figures and Tables

**Table 1:** Description of all environmental variables.

Description		
Climate	bio1	Annual mean temperature
	bio2	Mean diurnal range (mean of monthly (max temp − min temp))
	bio3	Isothermality (bio2/bio7) (×100)
	bio4	Temperature seasonality (standard deviation × 100)
	bio5	Max temperature of warmest month
	bio6	Min temperature of coldest month
	bio7	Temperature annual range (bio5–bio6)
	bio8	Mean temperature of wettest quarter
	bio9	Mean temperature of driest quarter
	bio10	Mean temperature of warmest quarter
	bio11	Mean temperature of coldest quarter
	bio12	Annual precipitation
	bio13	Precipitation of wettest month
	bio14	Precipitation of driest month
	bio15	Precipitation seasonality (coefficient of variation)
	bio16	Precipitation of wettest quarter
	bio17	Precipitation of driest quarter
	bio18	Precipitation of warmest quarter
	bio19	Precipitation of coldest quarter
Land	elevation	Elevation
	aspect	Aspect
	slope	Slope
Soil	t_cec_clay	Topsoil CEC (clay)
	t_cec_soil	Topsoil CEC (soil)
	t_caco3	Topsoil calcium carbonate
	t_esp	Topsoil Spdicity (ESP)
	t_ece	Topsoil Spdicity (Elco)
	t_oc	Topsoil organic carbon
	t_ph_h2o	Topsoil pH (H2O)
	t_ref_bulk	Topsoil reference bulk density
	t_usda_tex	Topsoil USDA texture classification

**Table 2:** Contribution percentage and significance of permutations of environmental factors that affect the distribution of species.

Code	Percent Contribution (%)	Permutation Importance (%)
bio6	43.29	26.77
t_cec_soil	15.82	0.76
bio7	12.65	23.28
bio19	6.92	8.81
elevation	5.89	11.51
bio9	5.24	10.03
bio14	2.93	5.10
bio18	2.41	3.21
bio16	1.60	6.71
bio15	1.46	2.50
t_cec_clay	1.09	0.14
bio8	0.70	1.18

## Data Availability

Data are contained within the article.
